# Graph regularized *L*_2,1_-nonnegative matrix factorization for miRNA-disease association prediction

**DOI:** 10.1186/s12859-020-3409-x

**Published:** 2020-02-18

**Authors:** Zhen Gao, Yu-Tian Wang, Qing-Wen Wu, Jian-Cheng Ni, Chun-Hou Zheng

**Affiliations:** School of Software, Qufu Normal University, Qufu, 273165 China

**Keywords:** miRNA, Disease, miRNA-disease associations, NMF *L*_2, 1_-norm

## Abstract

**Background:**

The aberrant expression of microRNAs is closely connected to the occurrence and development of a great deal of human diseases. To study human diseases, numerous effective computational models that are valuable and meaningful have been presented by researchers.

**Results:**

Here, we present a computational framework based on graph Laplacian regularized ***L***_2, **1**_-nonnegative matrix factorization (***GRL***_2, **1**_-NMF) for inferring possible human disease-connected miRNAs. First, manually validated disease-connected microRNAs were integrated, and microRNA functional similarity information along with two kinds of disease semantic similarities were calculated. Next, we measured Gaussian interaction profile (GIP) kernel similarities for both diseases and microRNAs. Then, we adopted a preprocessing step, namely, weighted K nearest known neighbours (WKNKN), to decrease the sparsity of the miRNA-disease association matrix network. Finally, the ***GRL***_2**,1**_-NMF framework was used to predict links between microRNAs and diseases.

**Conclusions:**

The new method (GRL_2, 1_-NMF) achieved AUC values of 0.9280 and 0.9276 in global leave-one-out cross validation (global LOOCV) and five-fold cross validation (5-CV), respectively, showing that GRL_2, 1_-NMF can powerfully discover potential disease-related miRNAs, even if there is no known associated disease.

## Background

MicroRNAs (miRNAs), which play crucial roles in the regulation of gene expression after transcription in organisms and vegetation, are 17–24 nt noncoding endogenous RNAs [1–3]. In 1993, Lee et al. [4] identified the first microRNA (miRNA) called lin-4 in *Caenorhabditis elegans*. Thereafter, a large number of miRNAs have been identified from a wide variety of species, such as plants, animals, and viruses [5, 6]. MiRNAs are associated with key biological processes, including development, differentiation, programmed cell death and cell proliferation [7, 8]. Past studies have indicated that abnormal miRNA expression participates in the development process of a variety of human diseases [9–11]. However, inferring microRNA-disease connections through manual experiments is tremendously costly, laborious, prone to failure and time consuming. Thus, the development of computation-based methods to infer disease-connected microRNAs is urgently needed, as they could solve the above problems and greatly facilitate human disease diagnosis and treatment [12–15].

For the past few years, in order to explore the pathogenic mechanism of human disease at the small molecule level and design specific molecular instruments for diagnosis treatment and prevention, considerable efforts have been made to develop computational algorithms for inferring disease-associated microRNAs according to the assumptions that microRNAs have similar functions that are highly likely to be connected with similar diseases, and vice versa. Numerous similarity measurement-based approaches according to heterogeneous biological information have been proposed to identify the interactions between microRNAs and diseases. Jiang et al. [16] inferred disease-related miRNAs by prioritizing the whole human miRNAome connected with disease that we investigated based on miRNA functional similarity information as well as the human phenome-microRNAome network. Li et al. [17] proposed a computation-based model to infer the possible disease–related miRNAs via calculations of FCS between the disease-gene and the target-gene, which had verification. There is an assumption that if two various diseases have phenotypic connections, they have similar molecular machinery and similar molecular mechanisms. Xu et al. [18] inferred human disease-connected microRNAs by fusing experimentally verified human disease genes as well as context-dependent miRNA-target interactions to prioritize disease-connected microRNAs. In line with weighted k nearest neighbours, HDMP was proposed by Xuan et al. [19] for identifying potential miRNA-disease associations. They presented a measurement method including the details of the disease term along with phenotypic similarities among diseases for the purpose of measuring the miRNA functional similarities. In addition, considering the miRNAs of the same miRNA family or cluster and their relationship to a group of diseases, they were given a higher weight. However, HDMP is not appropriate for diseases that have sparse connections with miRNAs. Chen et al. [20] developed miRPD in which experimentally verified or predicted interactions between miRNAs and proteins as well as text-extracted connections between protein and disease associations were explicitly utilized to calculate the probability that a microRNA-disease association exists. Chen et al. [21] developed WBSMDA according to the calculation of the within-scores and between-scores of every miRNA-disease group to identify potential disease-related miRNAs. Take a miRNA as an example, there is a miRNA set *A* whose elements all have known connections with the investigated disease *d*. The propose of within-scores is finding a miRNA in set *A* that has the highest similarity score with the investigated miRNA. There is a miRNA set *B* whose elements all have unknown connections with the investigated disease *d*. The proposed between-scores involves finding a miRNA in set *B* that has the highest similarity score with the investigated miRNA. Chen et al. [22] developed HGIMDA through an iteration approach in line with a graph that consists of many different types of bioinformatics information, such as the functional similarities of microRNAs, semantic similarities of diseases, kernel similarity of Gaussian interaction profiles and experimental verification of microRNA-disease connections. Yu et al. [23] proposed an assembled identification approach to infer potential microRNA-disease associations by modifying the existing maximizing information flow methods based on integrated microRNA functional similarity information, disease semantic information and phenotypic similarity information; these potential associations along with manually validated microRNA-disease interactions were placed into a phenome-microRNAome network. Chen et al. [24] presented a novel framework called RKNNMDA that utilizes ranking and k nearest neighbours. They integrated the functional similarity of microRNAs, semantic similarity of diseases, kernel similarity of Gaussian interaction profiles and experimental verification of microRNA-disease association and obtained miRNA’s (disease’s) k nearest neighbours via the KNN model. Next, they implemented the SVM ranking model to re-rank the above k nearest neighbours and thus obtained the eventual rankings of all possible microRNA-disease associations. In addition, RKNNMDA could also predict possible microRNA-disease connections for human diseases that don’t have manually validated associated miRNAs. Chen et al. [25] introduced Jaccard similarity among microRNAs and diseases in the BLHARMDA model to identify potential miRNA-disease interactions and then introduced an improved KNN framework into the bipartite local model method. Chen et al. [26] defined all paths between a given miRNA and disease as prediction scores, based on the assumption that if there are more paths between the miRNA and disease, the two are more likely to be related.

In addition, a host of studies in accordance with random walk with restart have been proposed for identifying potential microRNA-disease connections and finally obtained good predictive behaviour. A random walk with restart was presented by Chen et al. [27], who also integrated the manually verified microRNA-disease association information and functional similarity information of miRNAs. Considering the functional links among microRNA targets and human disease genes in a protein association network, Shi et al. [28] devised a computational model to infer likely microRNA-disease connections. This method utilized global network distance measurement, random walk analysis, and the construction of a microRNA-disease network to investigate microRNA-disease connections from a global perspective. Xuan et al. [29] designed a novel framework named MIDP, which predicted disease-connected miRNAs for diseases with known associated microRNAs in line with random walks. They analysed the attributes of the labelled and unlabelled nodes of the miRNA network and then established transition matrices, whose transition weights between the nodes were proportionate to the similarity between them. Furthermore, they presented an extension method called MIDPE, especially for diseases that don’t have manually verified connected microRNAs. Liu et al. [30] proposed a method to identify possible disease-connected microRNAs by utilizing a random walk with restart in accordance with a heterogeneous graph, which was established by combining disease semantic similarities and disease functional similarities, as well as the miRNA similarities that were obtained utilizing microRNA-target gene and microRNA-long noncoding RNA connections. Luo and Xiao [13] first established a heterogeneous network containing microRNA and disease information and then adopted a bi-random walk model to identify possible microRNA-disease connections. Finally, all microRNA candidates of an investigated disease were ranked.

Furthermore, machine learning-based algorithms, such as support vector machines, have been applied to bioinformatics and computational biology and have improved the prediction performance to some extent [31]. Xu et al. [32] presented MTDN to infer potential microRNA-disease associations. They identified positive disease-related miRNAs from negative samples through the SVM classifier in accordance with the characteristics of microRNA target-dysregulated network topology information. Chen et al. [33] identified miRNA-disease links based on regularized least squares (RLS) for identifying the miRNA-disease links. RLSMDA integrates known disease-microRNA connections, a disease semantic similarities dataset, and a miRNA functional similarities network and is thus suitable for predicting novel miRNAs for diseases without any manually validated connections with microRNAs. Li et al. [34] utilized a matrix completion model in line with manually validated microRNA-disease connections to infer candidates for diseases that did not have any experimentally proven connected microRNAs. In addition, MCMDA does not need negative associations. Chen et al. [35] proposed a random forest-based framework (RFMDA) for microRNA-disease connection prediction. RFMDA identifies possible disease-associated microRNAs by employing the random forest model to identify robust attributes from the miRNA-disease attribute collection. Chen et al. [36] predicted disease-associated miRNAs based on heterogeneous label propagation (HLPMDA), in which heterogeneous data were integrated into a heterogeneous network. Chen et al. [37] inferred disease-associated miRNAs with restricted Boltzmann machine (RBM); this model can acquire both disease-connected miRNAs as well as the corresponding forms of their links. However, this method is not suitable for diseases that do not have any known miRNA-disease associations, and selecting the right parameter values remains a significant issue for RBMMMDA. Chen et al. [38] first integrated a heterogeneous network, then put it into a stacked autoencoder for the purpose of detecting the deep representation of the heterogeneous information, finally utilizing an SVM classifier to prioritize all the candidates. Chen et al. [39] first constructed a feature vector according to the statistics, graph theory and matrix decomposition of the bioinformatics data and then put this vector into EGBMMDA to obtain a regression tree. Chen et al. [40] extracted three kinds of features, namely, statistical features from similarity measurements, graph theoretical features from similarity networks, and matrix factorization results from miRNA-disease associations. Then, disease-related miRNAs were discovered based on a decision tree classifier. Chen et al. [41] predicted disease-connected miRNAs by adopting sparse subspace learning with Laplacian regularization and *L*_1_-norm. Interestingly, they extracted features and constructed objective functions from miRNA and disease perspectives, separately. Chen et al. [42] used a decision tree as a weak classifier and then integrated these weak classifiers into a strong classifier according to weights. It is worth noting that they implemented k-means to balance positive samples and negative samples.

Moreover, many researchers have made promising models with recommendation systems for microRNA-disease connection prediction purposes. Zou et al. [43] proposed two approaches, namely, KATZ and CATAPULT, for identifying miRNA-disease links. In line with the manually verified microRNA-disease link network, microRNA similarities network and disease similarities network, KATZ integrates the social network analysis approach with machine learning. Chen et al. [44] inferred disease-related miRNAs based on ensemble learning and link prediction (ELLPMDA). According to global similarity measures, ELLPMDA uses ensemble learning for integrating ranking results, which were obtained via three typical similarity-measurement approaches. Chen et al. [45] constructed a heterogeneous network and predicted disease-connected miRNAs in line with the rating-integrated bipartite network recommendation as well as experimentally verified miRNA–disease connections.

In addition, a fair number of studies based on matrix factorization have been presented for possible disease-connected microRNA prediction purposes. Zhao et al. [46] presented symmetric nonnegative matrix factorization (SNMFMDA) to infer disease-connected microRNAs with the NMF and Kronecker regularized least square (KronRLS) approaches. Zhong et al. [47] proposed a nonnegative matrix factorization (NMF)-based algorithm to predict disease-related microRNA candidates based on a bilayer network that was constructed with regard to the intricate links among microRNAs, among human diseases and between microRNAs and human diseases. Xiao et al. [48] introduced graph Laplacian regularized into NMF (GRNMF) based on heterogeneous data for inferring potential disease-connected microRNAs, particularly for many diseases without known associations. They introduced a pre-processing step, weighted k nearest neighbour (WKNKN) profiles, for both microRNAs and diseases, into GRNMF. Chen et al. [49] designed an effective algorithm, MDHGI, according to matrix decomposition as well as a heterogeneous graph inference method for inferring potential miRNA-disease connections.

However, these approaches based on matrix factorization ignored the sparsity of the miRNA-disease association matrix *Y*, so we utilized a pre-processing step named weighted K nearest known neighbours (WKNKN) [50] to convert the value of the miRNA-disease associations matrix *Y* into a decimal between 0 and 1. In addition, unlike the traditional nonnegative matrix factorization (NMF) methods, we added *L*_2, 1_-norm as well as GIP (Gaussian interaction profile) kernels into the NMF model. The *L*_2, 1_-norm was added to increase the disease matrix sparsity and eliminate unattached disease pairs [51–53]. Moreover, Tikhonov regularization was added to penalize the non-smoothness of *W* and *H* [48, 54, 55], and the graph regularization was primarily intended to assure local-based representation by leveraging the geometry of the data [56].

In this study, we present a computational algorithm based on graph regularized *L*_2, 1_-nonnegative matrix factorization (*GRL*_2, 1_-NMF) to infer the possible connections between microRNAs and diseases in heterogeneous omics data. First, we integrated manually validated microRNA-disease connection information, miRNA functional similarity information and two kinds of disease semantic similarity information, and then we calculated the GIP kernel similarities for the diseases and miRNAs. Then, we utilized WKNKN to decrease the sparsity of matrix *Y*. Furthermore, we added Tikhonov (*L*_2_), graph Laplacian regularization terms and the *L*_2, 1_-norm to the standard NMF model for predicting disease-associated miRNAs. Finally, five-fold cross validation and global leave-one-out cross validation were implemented to evaluate the effectiveness of our model, and we obtained AUCs of 0.9276 and 0.9280, respectively. Furthermore, we performed case studies on three high-risk human diseases (prostate neoplasms, lung neoplasms and breast neoplasms). As a result, 48, 45 and 45 out of the top 50 likely connected miRNAs of prostate neoplasms, lung neoplasms and breast neoplasms, respectively, were confirmed by HMDD [10] and dbDEMC [57]. Based on the experimental results, we can clearly see that *GRL*_2, 1_-NMF is a valuable approach for inferring possible miRNA-disease connections.

## Results

### **Effect of parameters on the performance of*GRL***_**2**, **1**_-NMF

In this work, we measured two disease semantic similarities, miRNA functional similarity and GIP similarities for miRNAs and diseases. These two disease semantic similarities were integrated as Eq. (1), and the final disease similarity and miRNA similarity were measured as Eq. (2) and Eq. (3), respectively. We defined six parameters, namely, *α*_1_, *α*_2_, *γ*_1_, *γ*_2_, *θ*_1_ and *θ*_2_, to balance the items in Eq. (1), Eq. (2) and *Eq.* (3). The values of *α*_1_ and *α*_2_ ranged from 0.1, 0.2, 0.3, ... to 0.9. *γ*_1_, *γ*_2_, *θ*_1_ and *θ*_2_ ranged from 0,0.1,0.2, ... 0.9, to 1. We conducted a series of experiments on the above parameters to acquire the effects of these parameters. The experimental results are shown in Table 1 and Table 2.

1 $$ SD1\left({d}_i,{d}_j\right)={\alpha}_1{S}_1^d\left({d}_i,{d}_j\right)+{\alpha}_2{S}_2^d\left({d}_i,{d}_j\right) $$

2 $$ SD\left({d}_i,{d}_j\right)=\left\{\begin{array}{cc}{\gamma}_1 SD1\left({d}_i,{d}_j\right)+{\gamma}_2 GD\left({d}_i,{d}_j\right)& {d}_i\  and\ {d}_j\  have\ semantic\ similarity\\ {} GD\left({d}_i,{d}_j\right)& otherwise\end{array}\right. $$

3 $$ SM\left({m}_i,{m}_j\right)=\left\{\begin{array}{cc}{\theta}_1{S}^m\left({m}_i,{m}_j\right)+{\theta}_2 GM\left({m}_i,{m}_j\right)& {m}_i\  and\ {m}_j\  have\ functional\ similarity\\ {} GM\left({m}_i,{m}_j\right)& otherwise\end{array}\right. $$

**Table 1:** The effects of parameters ***α***_**1**_ and ***α***_**2**_ on the results of *GRL*_2, 1_-NMF ***γ***_**1**_ ***=*** **1,*γ***_**2**_ ***=*** **0,** ***θ***_**1**_ ***=*** **1,and** ***θ***_**2**_ ***=*** **0**

	*α*_1_	*α*_2_	AUCs of 5-CV
SD12_1	0.1	0.9	0.9276
SD12_2	0.2	0.8	0.9276
SD12_3	0.3	0.7	0.9276
SD12_4	0.4	0.6	0.9276
SD12_5	0.5	0.5	0.9276
SD12_6	0.6	0.4	0.9276
SD12_7	0.7	0.3	0.9276
SD12_8	0.8	0.2	0.9276
SD12_9	0.9	0.1	0.9276

**Table 2:** The effects of parameters ***θ***_**1**_**,*θ***_**2**_**,** ***γ***_**1**_**, and** ***γ***_**2**_ on the results of *GRL*_2, 1_-NMF (a) ***α***_**1**_ = **0.5**, ***α***_**2**_ = **0.5**, ***γ***_**1**_ = **1**, and ***γ***_**2**_ = **0** (b) ***α***_**1**_ **= 0.5,*α***_**2**_ ***=*** **0.5,** ***θ***_**1**_ ***=*** **1,and** ***θ***_**2**_ ***=*** **0**

	***θ***_**1**_	***θ***_**2**_	AUCs of 5-CV		***γ***_**1**_	***γ***_**2**_	AUC of 5-CV
SMGM_1	0.1	0.9	0.9263	SDGD_1	0.1	0.9	0.9276
SMGM_2	0.2	0.8	0.9264	SDGD_2	0.2	0.8	0.9276
SMGM_3	0.3	0.7	0.9267	SDGD_3	0.3	0.7	0.9276
SMGM_4	0.4	0.6	0.9268	SDGD_4	0.4	0.6	0.9276
SMGM_5	0.5	0.5	0.9270	SDGD_5	0.5	0.5	0.9276
SMGM_6	0.6	0.4	0.9270	SDGD_6	0.6	0.4	0.9276
SMGM_7	0.7	0.3	0.9271	SDGD_7	0.7	0.3	0.9276
SMGM_8	0.8	0.2	0.9272	SDGD_8	0.8	0.2	0.9276
SMGM_9	0.9	0.1	0.9272	SDGD_9	0.9	0.1	0.9276
SMGM_10	1	0	0.9276	SDGD_10	1	0	0.9276

In Table 1, we can see that regardless of how α_1_ and α_2_ change, the AUC of 5-CV remains 0.9276. Thus, for convenience, we set *α*_1_ = *α*_2_ = 0.5*.* The experimental results of parameters *θ*_1_ and *θ*_2_ that balanced miRNA functional similarity (*S*^*m*^) and GIP similarity for miRNAs (GM) are shown in Table 2 (a), and the results of parameters *γ*_1_ and *γ*_2_ that balanced disease semantic similarity (*SD*1) and GIP similarity for diseases (GD) are shown in Table 2 (b). Thus, we set *θ*_1_ = 1, *θ*_2_ = 0, *γ*_1_ = 1, and *γ*_2_ = 0.

### Performance evaluation

To evaluate our model’s ability to predict disease-related miRNAs, we compared it with three state-of-art methods (ICFMDA [58], SACMDA [59] and IMCMDA [60]) by implementing two validation frameworks: global leave-one-out cross validation (global LOOCV) and five-fold cross validation (5-CV) according to the experimentally validated disease-related miRNAs in HMDD v2.0, which gathered plenty of the known miRNA-disease associations [10].

**Fig. 1:**
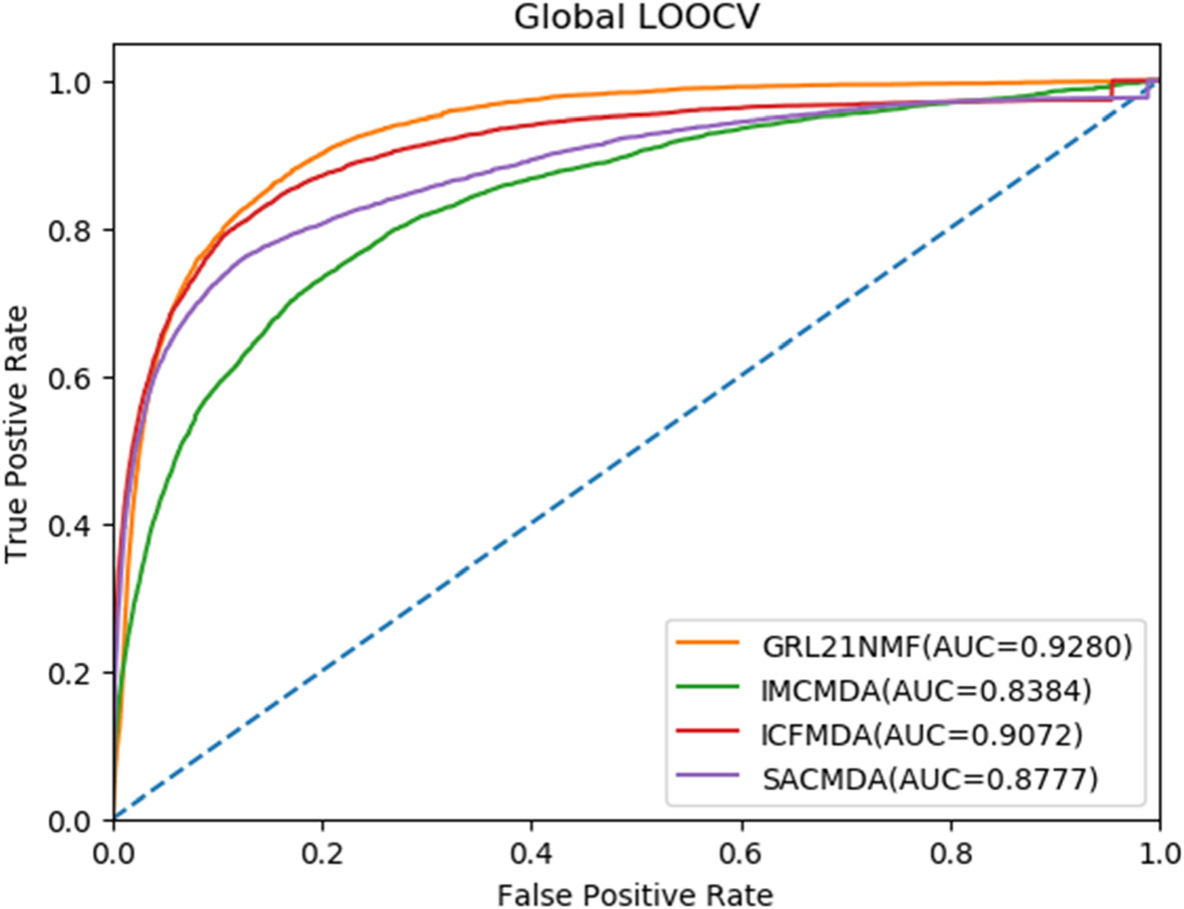
AUC of global LOOCV compared with those of IMC, ICFMDA and SACMDA

For the global LOOCV, every known miRNA-disease connection was selected in turn for testing, and others that had also been experimentally verified were considered as training sets for the purpose of model training. In addition, all miRNA-disease associations without evidence were regarded as candidate samples. Next, we calculated the prediction score of all associations by implementing *GRL*_2, 1_-NMF and thus obtained the ranking of each test sample compared with that of the candidate samples. We hold our model as efficient if the ranking of each test sample was higher than a certain threshold. We obtained the corresponding true positive rate (TPR, sensitivity) and false positive rate (FPR, 1-specificity) by setting various thresholds. Sensitivity is the proportion of the testing samples whose ranking was higher than the threshold, while 1-specificity calculates the percentage of the testing samples whose ranking was lower than the threshold. Thus, the receiver operating characteristic (ROC) curve can be plotted in line with TPRs and FPRs obtained by different thresholds. Finally, to evaluate the performance and compare it with that of the other models, the areas under the ROC curve (AUCs) were computed. The AUC value is between 0 and 1, and a model whose AUC value is higher has a better performance. The results showed that *GRL*_2, 1_-NMF, ICFMDA, SACMDA and IMCMDA achieved AUC values of 0.9280, 0.9072, 0.8777 and 0.8384, respectively (see Fig. 1). Clearly, *GRL*_2, 1_-NMF obtained the best performance among the four explored methods.

**Fig. 2:**
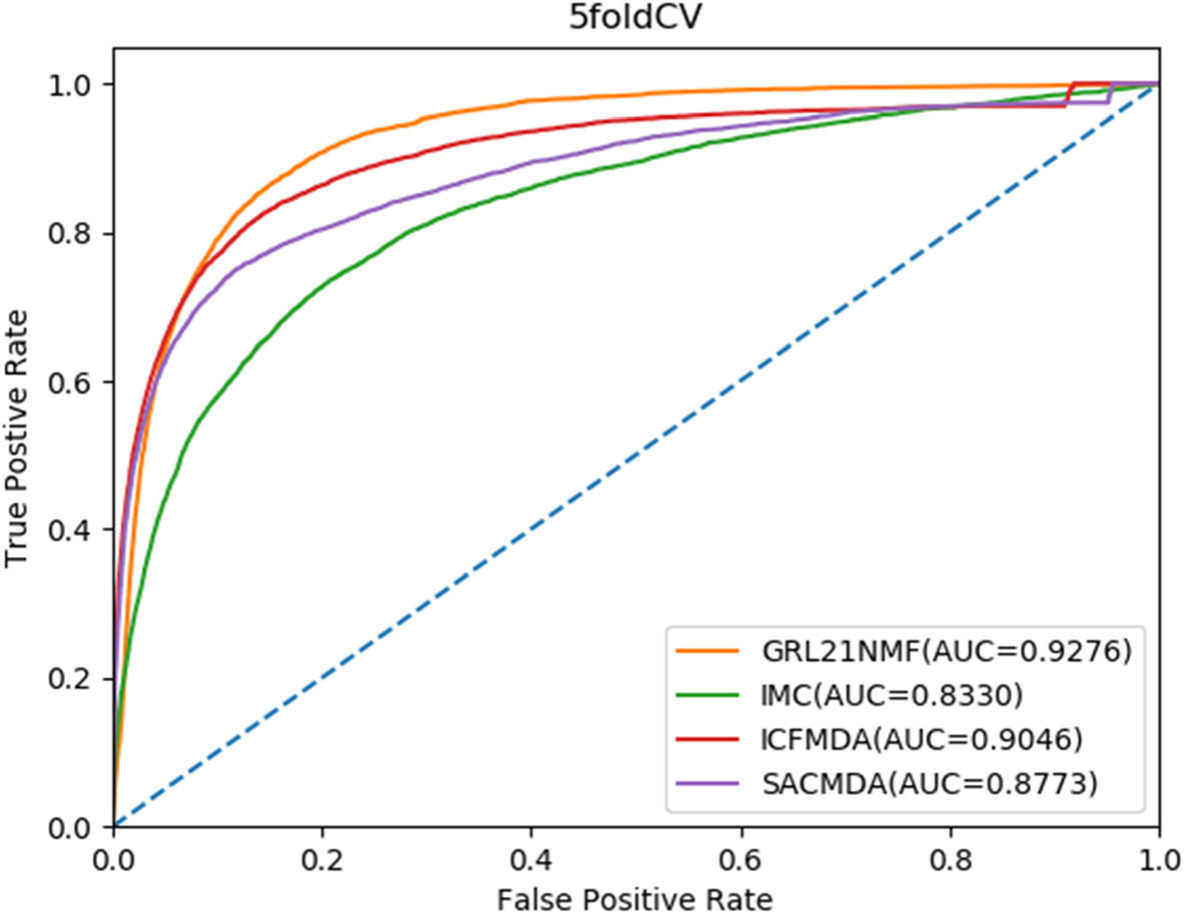
AUC of 5-fold cross validation compared with those of IMC, ICFMDA and SACMDA

For 5-CV, all known connections between microRNAs and diseases were randomly distributed into five parts, where one part was selected in turn for testing, and the other four parts were used in turn for training. Moreover, all unknown samples were treated as candidate samples. Like the global LOOCV, we finally calculated the ranking of the test sample relative to the candidate set. Considering the possible bias caused by random sample partitioning for performance evaluation, we repeatedly divided the known miRNA disease associations 100 times and obtained the corresponding ROC curves and AUCs in a similar manner to that for LOOCV. The results showed that *GRL*_2, 1_-NMF had the best predictive performance with an average AUC of 0.9276, and ICFMDA, SACMDA and IMCMDA achieved AUC values of 0.9046, 0.8773 and 0.8330, respectively (see Fig. 2).

### Case studies

We constructed a simulation experiment to further demonstrate the effectiveness of *GRL*_2, 1_-NMF for inferring likely disease-connected miRNAs. Here, all manually validated miRNA-disease connections were utilized for prediction, and other associations that did not have evidence were regarded as candidate connections for validation. For every disease, the candidate miRNAs were ranked based on the prediction scores. We used two miRNA-disease databases, namely, HMDD [10] and dbDEMC [57], to verify the inferred possible microRNAs for the investigated disease, including prostate neoplasms, breast neoplasms and lung neoplasms. Finally, the top 50 disease-related miRNAs predicted via *GRL*_2, 1_-NMF are demonstrated in Table 3, Table 4 and Table 5. There are 48,45 and 45 of 50 inferred miRNAs confirmed to have associations with prostate neoplasms, breast neoplasms and lung neoplasms, respectively, by the dbDEMC database and HMDD v3.0 database.

**Table 3:** The top 50 potential miRNAs associated with Prostate Neoplasms

miRNA	Evidence	miRNA	Evidence
hsa-mir-1	HMDD; dbDEMC	hsa-mir-32	HMDD; dbDEMC
hsa-mir-21	HMDD; dbDEMC	hsa-let-7i	dbDEMC
hsa-mir-22	HMDD; dbDEMC	hsa-mir-375	HMDD; dbDEMC
hsa-mir-155	HMDD; dbDEMC	hsa-let-7c	HMDD; dbDEMC
hsa-mir-9	HMDD	hsa-mir-200c	HMDD; dbDEMC
hsa-mir-221	HMDD; dbDEMC	hsa-mir-214	HMDD; dbDEMC
hsa-let-7a	dbDEMC	hsa-mir-182	HMDD; dbDEMC
hsa-mir-133a	HMDD; dbDEMC	hsa-mir-106b	HMDD; dbDEMC
hsa-mir-146a	HMDD	hsa-mir-23a	HMDD; dbDEMC
hsa-mir-222	HMDD; dbDEMC	hsa-mir-17	HMDD; dbDEMC
hsa-mir-34a	HMDD; dbDEMC	hsa-let-7e	dbDEMC
hsa-mir-29a	HMDD; dbDEMC	hsa-mir-181	unconfirmed
hsa-mir-142	unconfirmed	hsa-mir-200b	HMDD; dbDEMC
hsa-mir-223	HMDD; dbDEMC	hsa-mir-10b	dbDEMC
hsa-mir-126	HMDD; dbDEMC	hsa-mir-200a	HMDD; dbDEMC
hsa-mir-31	HMDD; dbDEMC	hsa-mir-34c	HMDD
hsa-mir-146b	HMDD; dbDEMC	hsa-mir-205	HMDD; dbDEMC
hsa-mir-29b	HMDD; dbDEMC	hsa-let-7d	HMDD; dbDEMC
hsa-mir-200	HMDD	hsa-mir-210	HMDD; dbDEMC
hsa-mir-143	HMDD; dbDEMC	hsa-mir-192	HMDD; dbDEMC
hsa-mir-16	HMDD; dbDEMC	hsa-mir-196a	HMDD; dbDEMC
hsa-mir-20a	HMDD; dbDEMC	hsa-mir-195	HMDD; dbDEMC
hsa-mir-30a	HMDD	hsa-let-7f	dbDEMC
hsa-let-7b	HMDD; dbDEMC	hsa-mir-181b	HMDD; dbDEMC
hsa-mir-199a	HMDD; dbDEMC	hsa-mir-34b	HMDD

**Table 4:** The top 50 potential miRNAs associated with Lung Neoplasms

miRNA	Evidence	miRNA	Evidence
hsa-mir-1	HMDD	hsa-mir-139	HMDD; dbDEMC
hsa-mir-181	unconfirmed	hsa-mir-193b	dbDEMC
hsa-mir-200	HMDD	hsa-mir-204	dbDEMC
hsa-mir-26	HMDD	hsa-mir-708	dbDEMC
hsa-mir-195	dbDEMC	hsa-mir-378a	unconfirmed
hsa-mir-92	dbDEMC	hsa-mir-625	dbDEMC
hsa-mir-141	dbDEMC	hsa-mir-367	dbDEMC
hsa-mir-122	HMDD; dbDEMC	hsa-mir-149	HMDD; dbDEMC
hsa-mir-16	HMDD; dbDEMC	hsa-mir-148b	HMDD; dbDEMC
hsa-mir-99a	HMDD; dbDEMC	hsa-mir-328	HMDD; dbDEMC
hsa-mir-129	HMDD; dbDEMC	hsa-mir-302b	dbDEMC
hsa-mir-429	dbDEMC	hsa-mir-302a	dbDEMC
hsa-mir-130a	HMDD; dbDEMC	hsa-mir-373	HMDD; dbDEMC
hsa-mir-451	HMDD; dbDEMC	hsa-mir-92b	dbDEMC
hsa-mir-451a	HMDD; dbDEMC	hsa-mir-23b	dbDEMC
hsa-mir-15b	dbDEMC	hsa-mir-152	HMDD; dbDEMC
hsa-mir-151	unconfirmed	hsa-mir-196b	HMDD; dbDEMC
hsa-mir-15a	HMDD; dbDEMC	hsa-mir-302c	dbDEMC
hsa-mir-151a	unconfirmed	hsa-mir-452	dbDEMC
hsa-mir-296	unconfirmed	hsa-mir-215	HMDD; dbDEMC
hsa-mir-320a	dbDEMC	hsa-mir-302d	dbDEMC
hsa-mir-20b	dbDEMC	hsa-mir-28	dbDEMC
hsa-mir-342	HMDD; dbDEMC	hsa-mir-520a	dbDEMC
hsa-mir-194	HMDD; dbDEMC	hsa-mir-130b	HMDD; dbDEMC
hsa-mir-106b	dbDEMC	hsa-mir-372	HMDD; dbDEMC

**Table 5:** The top 50 potential miRNAs associated with Breast Neoplasms

miRNA	Evidence	miRNA	Evidence
hsa-mir-1	HMDD; dbDEMC	hsa-mir-330	dbDEMC
hsa-mir-32	HMDD; dbDEMC	hsa-mir-192	HMDD; dbDEMC
hsa-mir-106a	HMDD; dbDEMC	hsa-mir-28	dbDEMC
hsa-mir-26	unconfirmed	hsa-mir-130b	HMDD; dbDEMC
hsa-mir-99a	HMDD; dbDEMC	hsa-mir-211	dbDEMC
hsa-mir-151	HMDD; dbDEMC	hsa-mir-181c	HMDD; dbDEMC
hsa-mir-451	HMDD; dbDEMC	hsa-mir-449a	HMDD; dbDEMC
hsa-mir-92	HMDD; dbDEMC	hsa-mir-449b	dbDEMC
hsa-mir-130a	HMDD; dbDEMC	hsa-mir-99b	dbDEMC
hsa-mir-15b	HMDD; dbDEMC	hsa-mir-208a	HMDD; dbDEMC
hsa-mir-150	HMDD; dbDEMC	hsa-mir-650	dbDEMC
hsa-mir-185	HMDD; dbDEMC	hsa-mir-491	HMDD
hsa-mir-142	HMDD	hsa-mir-532	unconfirmed
hsa-mir-378a	HMDD	hsa-mir-144	HMDD; dbDEMC
hsa-mir-186	dbDEMC	hsa-mir-181d	dbDEMC
hsa-mir-95	dbDEMC	hsa-mir-494	HMDD; dbDEMC
hsa-mir-92b	HMDD; dbDEMC	hsa-mir-362	unconfirmed
hsa-mir-196b	HMDD; dbDEMC	hsa-mir-517a	dbDEMC
hsa-mir-98	HMDD; dbDEMC	hsa-mir-371	dbDEMC
hsa-mir-372	dbDEMC	hsa-mir-371a	unconfirmed
hsa-mir-574	HMDD	hsa-mir-381	HMDD; dbDEMC
hsa-mir-542	unconfirmed	hsa-mir-216a	dbDEMC
hsa-mir-370	HMDD; dbDEMC	hsa-mir-433	dbDEMC
hsa-mir-212	HMDD; dbDEMC	hsa-mir-134	HMDD; dbDEMC
hsa-mir-30e	HMDD	hsa-mir-376a	HMDD; dbDEMC

## Discussion

Our method, *GRL*_2, 1_-NMF, is an efficient tool for predicting miRNA-disease associations according to the experimental results. The main contributions of this study are listed. First, we added GIP kernel similarities for miRNA and disease associations into the similarity measurement, which improved the dataset reliability. Second, considering the sparsity of observed miRNA-disease associations, we performed a pre-processing step (WKNKN) to solve this problem, thus enhancing the prediction performance of our model. Third, as a common model of recommendation systems, NMF also plays a crucial role in bioinformatics. However, standard NMF did not achieve satisfactory performance. Therefore, we added the Tikhonov (*L*_2_), graph Laplacian regularization terms and the *L*_2, 1_-norm into the standard NMF, which makes this model more reliable and robust. Finally, the AUCs of *GRL*_2, 1_-NMF are higher than those of some excellent models.

Note that DNSGRMF [53], which also predicts miRNA-disease connections, is a graph regularized method similar to *GRL*_2, 1_-NMF. Both methods decompose the original matrix *Y* into two matrices *W* and *H*, and then we can acquire a recovery matrix *Y*^∗^ = *W* ∗ *H*. It is worth noting that *GRL*_2, 1_-NMF is based on nonnegative factorization, while DNSGRMF is based on graph regularized matrix factorization. DNSGRMF has no constraints, while *GRL*_2, 1_-NMF has two constraints of W ≥ 0 and H ≥ 0.

Nevertheless, our model still has room for improvement. First, miRNA information and disease information did not integrate perfectly, and we will improve this in future studies. Second, there may be more appropriate regularization terms that can improve the performance for miRNA-disease association prediction.

## Conclusions

It is meaningful and significant to predict disease-related miRNAs in studying the intrinsic aetiological factors of human diseases. A new model named *GRL*_2, 1_-NMF was developed in this work for potential miRNA-disease association prediction. First, we integrated experimentally validated connections between miRNAs and disease as well as miRNA functional similarities along with two kinds of disease semantic similarities, and then we calculated the GIP kernel similarities of microRNAs and diseases. Moreover, we used WKNKN to convert the value of matrix Y into a decimal between 0 and 1 and decrease the sparsity of matrix Y. Furthermore, the Tikhonov (*L*_2_), graph Laplacian regularization terms and the *L*_2, 1_-norm were added into the traditional NMF model for predicting miRNA-disease connections. In addition, the Tikhonov regularization was utilized to penalize the non-smoothness of *W* and *H*, and the graph Laplacian regularization was primarily intended to guarantee local-based representation by leveraging the geometric structure of the data. The *L*_2, 1_-norm was added to increase the disease matrix sparsity and eliminate unattached disease pairs.

Our method performs well in global LOOCV, 5-CV and case studies in heterogeneous omics data. The experimental results indicate that *GRL*_2, 1_-NMF can effectively and powerfully infer disease-related miRNAs, even if there are no known miRNA-disease associations. However, this method still has limitations that need further research. First, our similarity measurement for *GRL*_2, 1_-NMF might not be perfect, and other miRNA information still needs to be taken into account. Moreover, there is still room for improvement in the predictive performance of our method.

## Methods

### Human miRNA-disease associations

We collected information on all experimentally validated human miRNA-disease associations stored in the HMDD v2.0 database [10]. An adjacency matrix *Y* ∈ *R*^*n* × *m*^ was established to represent the manually verified human miRNA-disease associations, and the rows and columns of matrix *Y* represent miRNA *m*_*i*_ interactions and diseases *d*_*j*_ interactions, respectively. Therefore, in this study, the number of rows and columns in Y was 495 and 383, respectively. If a miRNA *m*_*i*_ has a known connection with a disease *d*_*j*_, *Y*_*ij*_ = 1, else *Y*_*ij*_ = 0.

### MiRNA functional similarity

There is a hypothesis that if two miRNAs are similar functionally, they are more likely to have connections with diseases that have high similarity, and vice versa [61, 62]. Wang et al. [63] shared their investigation results, and researchers can download miRNA functional similarity information at http://www.cuilab.cn/files/images/cuilab/misim.zip. Here, we established a matrix *S*^*m*^ that was denoted as the microRNA functional similarities. The item *S*^*m*^(*m*_*i*_, *m*_*j*_) denotes the functional similarities among microRNAs *m*_*i*_ and *m*_*j*_.

### Disease semantic similarity method 1

In this study, we take full advantage of the hierarchical directed acyclic graphs (DAGs) for disease similarity measurement based on the strategy of Wang et al. [63], and the disease DAG could be downloaded from the Medical Subject Headings (MeSH) database. *DAG*_*d*_ = (*d*, *T*_*d*_, *E*_*d*_) denotes the hierarchical DAG of disease d, where *T*_*d*_ denotes the disease collection, and *E*_*d*_ denotes links set in the DAG. According to the DAGs, the semantic values of disease D can be computed as Eq. (4).

4 $$ DV1(D)={\sum}_{d\in T(D)}D{1}_D(d) $$

where *D*1_*D*_(*d*) denotes the semantic contributions of disease *d’* to disease *d*, and *∆* denotes the semantic contribution factor (∆ = 0.5) [63].

5 $$ \left\{\begin{array}{c}D{1}_D(d)=1\  if\ d=D\\ {}D{1}_D(d)=\max \left\{\Delta  \ast D{1}_D\left({d}^{\prime}\right)|{d}^{\prime}\in child\ of\ d\right\} if\ d\ne D\end{array}\right. $$

Therefore, two diseases would likely have greater similarities if they share a larger part of their DAGs, and we can calculate semantic similarities between disease *d*_*i*_ and *d*_*j*_ as follows:

6 $$ {S}_1^d\left({d}_i,{d}_j\right)=\frac{\sum_{t\in T\left({d}_i\right)\cap T\left({d}_j\right)}\left(D{1}_{d_i}(t)+D{1}_{d_j}(t)\right)}{DV1\left({d}_i\right)+ DV1\left({d}_j\right)} $$

### Disease semantic similarity method 2

In the strategy for calculating disease semantic similarities above, diseases that shared one layer of *DAG*_*d*_ shared a common contribution value. However, if some diseases merely exist in fewer DAGs, then these diseases are called more specific diseases and should have a higher semantic contribution to disease *d*. In view of the algorithm presented by [19, 45], we can calculate the semantic contributions of disease *d* to disease *D* and the semantic values of disease *D* as Eq. (7) and Eq. (8), respectively.

7 $$ D{2}_D(d)=-\log \left(\frac{the\ number\ of\ DAGs\ including\ d}{the\ number\ of\ diseases}\right) $$

8 $$ DV2(D)={\sum}_{d\in T(D)}D{2}_D(d) $$

where d denotes any investigated disease. Finally, we could calculate the semantic similarities of diseases *d*_*i*_ and *d*_*j*_ as Eq. (9).

9 $$ {S}_2^d\left({d}_i,{d}_j\right)=\frac{\sum_{t\in T\left({d}_i\right)\cap T\left({d}_j\right)}\left(D{2}_{d_i}(t)+D{2}_{d_j}(t)\right)}{DV2\left({d}_i\right)+ DV2\left({d}_j\right)} $$

where the numerator of Equation (9) represents the common ancestor nodes of diseases *d*_*i*_ and *d*_*j*_, and the denominator denotes the entire ancestor nodes of diseases *d*_*i*_ and *d*_*j*_.

### Gaussian interaction profile kernel similarity for diseases and MiRNAs

If two diseases are similar, they are likely to have associations with microRNAs that are functionally approximate, and vice versa [61–64]. Gaussian interaction profile (GIP) kernel similarities have been adopted to quantify disease similarities and miRNA similarities [60, 65, 66]. We also calculated GIP kernel similarities for diseases and miRNAs in this work. First, based on whether disease *d*_*i*_(*m*_*j*_) has a known connection with each miRNA (disease) of the adjacency matrix *Y*, the interaction profiles *IP*(*d*_*i*_) and *IP*(*m*_*j*_) were constructed for disease *d*_*i*_ and miRNA *m*_*j*_, respectively. Then, the GIP kernel similarity between a disease pair and a miRNA pair is computed as Equation (10) and Equation (11), respectively.

10 $$ GD\left({d}_i,{d}_j\right)=\exp \left(-{\beta}_d{\left\Vert IP\left({d}_i\right)- IP\left({d}_j\right)\right\Vert}^2\right) $$

11 $$ GM\left({m}_i,{m}_j\right)=\exp \left(-{\beta}_m{\left\Vert IP\left({m}_i\right)- IP\left({m}_j\right)\right\Vert}^2\right) $$

Here, the kernel bandwidths *β*_*m*_ and *β*_*d*_ are described as Equation (12) and Equation (13), respectively, where ${\beta}_m^{\prime }$ and ${\beta}_m^{\prime }$ are both the original bandwidths.

12 $$ {\beta}_m={\beta}_m^{\prime }/\frac{1}{nm}{\sum}_{i=1}^{nm}{\left\Vert IP\left({m}_i\right)\right\Vert}^2 $$

13 $$ {\beta}_d={\beta}_d^{\prime }/\frac{1}{nd}{\sum}_{i=1}^{nd}{\left\Vert IP\left({d}_i\right)\right\Vert}^2 $$

In summary, the matrix *GD* and *GM* denote the GIP kernel similarity for diseases and miRNAs, respectively.

### Integrated similarity for diseases and MiRNAs

According to the various similarity measurement methods mentioned above, we combined the GIP kernel similarities with two disease semantic similarities as well as the miRNA functional similarities to obtain integrated disease similarities and integrated miRNA similarities, respectively. The weight setting problem of the above similarities is described in detail in the Results section, and we chose the following measurement strategy according to the experimental results. Specifically, if two miRNAs *m*_*i*_ and *m*_*j*_ had functional similarities, then the final similarity was the functional similarity. If two miRNAs *m*_*i*_ and *m*_*j*_ did not have functional similarities, then the final similarity was the GIP kernel similarity. Hence, the miRNA similarities score matrix *SM* between miRNA *m*_*i*_ and miRNA *m*_*j*_ is established as follows. Similarly, the disease similarity matrix *SD* is computed as follows:

14 $$ SM\left({m}_i,{m}_j\right)=\left\{\begin{array}{cc}{S}^m\left({m}_i,{m}_j\right)& {m}_i\  and\ {m}_j\  have\ functional\ similarity\\ {} GM\left({m}_i,{m}_j\right)& otherwise\end{array}\right. $$

15 $$ SD\left({d}_i,{d}_j\right)=\left\{\begin{array}{cc}\frac{S_1^d\left({d}_i,{d}_j\right)+{S}_2^d\left({d}_i,{d}_j\right)}{2}& {d}_i\  and\ {d}_j\  have\ semantic\ similarity\\ {} GD\left({d}_i,{d}_j\right)& otherwise\end{array}\right. $$

### Weighted K nearest known Neighbours (WKNKN) for MiRNAs and diseases

Let *M* = {*m*_1_, *m*_2_, …, *m*_*n*_} and *D* = {*d*_1_, *d*_2_, …, *d*_*m*_} represent the collection of *n* microRNAs and *m* diseases, respectively. We described the quantity of the investigated miRNAs and diseases as *n* and *m*, respectively, and then established an association matrix *Y* ∈ *R*^*n* × *m*^ to denote the known human microRNA-disease connections according to the HMDD v2.0 [10] database. If a miRNA *m*_*i*_ had been manually validated to be related to a disease *d*_*j*_, then *Y*_*ij*_ is equal to 1; otherwise, it is equal to 0. *Y*(*m*_*i*_) = {*Y*_*i*1_, *Y*_*i*2_, …, *Y*_*im*_}, namely, the *i*th row vector of matrix *Y*, represents the interaction profile for miRNA *m*_*i*_. Similarly, *Y*(*d*_*j*_) = {*Y*_1*j*_, *Y*_2*j*_, …, *Y*_*nj*_}, the *j*th column vector of matrix *Y*, represents the interaction profile for disease *d*_*j*_. In this study, we investigated 495(n) miRNAs and 383(m) diseases, yet the adjacency matrix *Y* ∈ *R*^*n* × *m*^ has merely 5430 known entries; thus, *Y* is a sparse matrix. Here, we performed a pre-processing procedure named weighted K nearest known neighbours (WKNKN) [50] for miRNAs and diseases without any known associations to resolve the abovementioned sparse problem and thus improve the prediction accuracy. After executing WKNKN, the entry *Y*_*ij*_ was replaced with a continuous value ranging from 0 to 1, and the specific steps are as follows.

First, we acquired the interaction profile of each miRNA *m*_*q*_ according to the functional similarity between *m*_*q*_ and its K nearest known miRNAs as follows:

16 $$ {Y}_m\left({m}_q\right)=\frac{1}{Q_m}{\sum}_{i=1}^K{w}_iY\left({m}_i\right) $$

where *m*_1_ to *m*_*K*_ are the miRNAs sorted in descending order based on their similarities to *m*_*q*_; *w*_*i*_ is the weight factor, and *w*_*i*_ = *α*^*i* − 1^ ∗ *S*^*m*^(*m*_*i*_, *m*_*q*_); in other words, the higher the similarity between *m*_*i*_ and *m*_*q*_ is, the higher the weight. *α* ∈ [0, 1] is a decay term, and *Q*_*m*_ = ∑_1 ≤ *i* ≤ *K*_*S*^*m*^(*m*_*i*_, *m*_*q*_) is the normalization coefficient.

Second, we acquired the interaction profile of each miRNA *d*_*p*_ according to the semantic similarity between *d*_*p*_ and its K nearest known diseases as follows:

17 $$ {Y}_d\left({d}_p\right)=\frac{1}{Q_d}{\sum}_{j=1}^K{w}_jY\left({d}_j\right) $$

where *d*_1_ to *d*_*K*_ are the diseases sorted in descending order based on their similarities to *d*_*p*_; *w*_*j*_ is the weight factor, and *w*_*j*_ = *α*^*j* − 1^ ∗ *S*^*d*^(*d*_*j*_, *d*_*p*_); in other words, the higher the similarity between *d*_*j*_ and *d*_*p*_ is, the higher weight. *Q*_*d*_ = ∑_1 ≤ *j* ≤ *K*_*S*^*d*^(*d*_*j*_, *d*_*p*_) is the normalization term.

Finally, we took the average of the above two values instead of *Y*_*ij*_ = 0, indicating the overall likelihood of the interaction between *m*_*i*_ and *d*_*j*_. Then, we integrated the above two matrices *Y*_*m*_ and *Y*_*d*_ acquired from different datasets, replaced *Y*_*ij*_ = 0 with the related likelihood scores, and then updated the original adjacency matrix *Y* as follows:

18 $$ {Y}_{md}={a}_1{Y}_m+{a}_2{Y}_d/\sum {a}_i\left(i=1,2\right) $$

19 $$ Y=\max \left(Y,{Y}_{md}\right) $$

where *a*_*i*_ is the weight coefficient and *a*_1_ = *a*_2_ = 1.

### Standard NMF

In recent years, as one of the common methods of recommendation systems, nonnegative matrix factorization (NMF) has been widely used as an effective prediction algorithm in the field of bioinformatics [67, 68]. Two non-negative matrices *W* and *H*, which are optimal approximations to the original matrix *Y*, can be found by NMF, where *W* and *H* satisfy Equation (20).

20 $$ \mathrm{Y}\approx \mathrm{W}{H}^T,\mathrm{s}.\mathrm{t}.W\ge 0,H\ge 0 $$

In this work, matrix *Y* ∈ *R*^*n* × *m*^ was used to represent the known miRNA-disease associations, and *NMF* can decompose this matrix into two matrices, namely, *W* ∈ *R*^*n* × *k*^ and *H* ∈ *R*^*m* × *k*^. Here, we express the question of the miRNA-disease association identification problem as the objective function (Equation (21)).

21 $$ {\mathit{\min}}_{W,H}{\left\Vert Y-W{H}^T\right\Vert}_F^2\ \mathrm{s}.\mathrm{t}.\mathrm{W}\ge 0,\mathrm{H}\ge 0 $$

where ‖∙‖_*F*_ represents the Frobenius norm of a matrix. Equation (21) can be optimized by taking advantage of the iterative update algorithm presented by [69].

However, standard NMF does not ensure the sparsity of decomposition; therefore, local-based representations are not always generated [70, 71]. Some researchers have developed sparse constraints on NMF [46–48].

### ***GRL***_**2**, **1**_-NMF

**Fig. 3:**
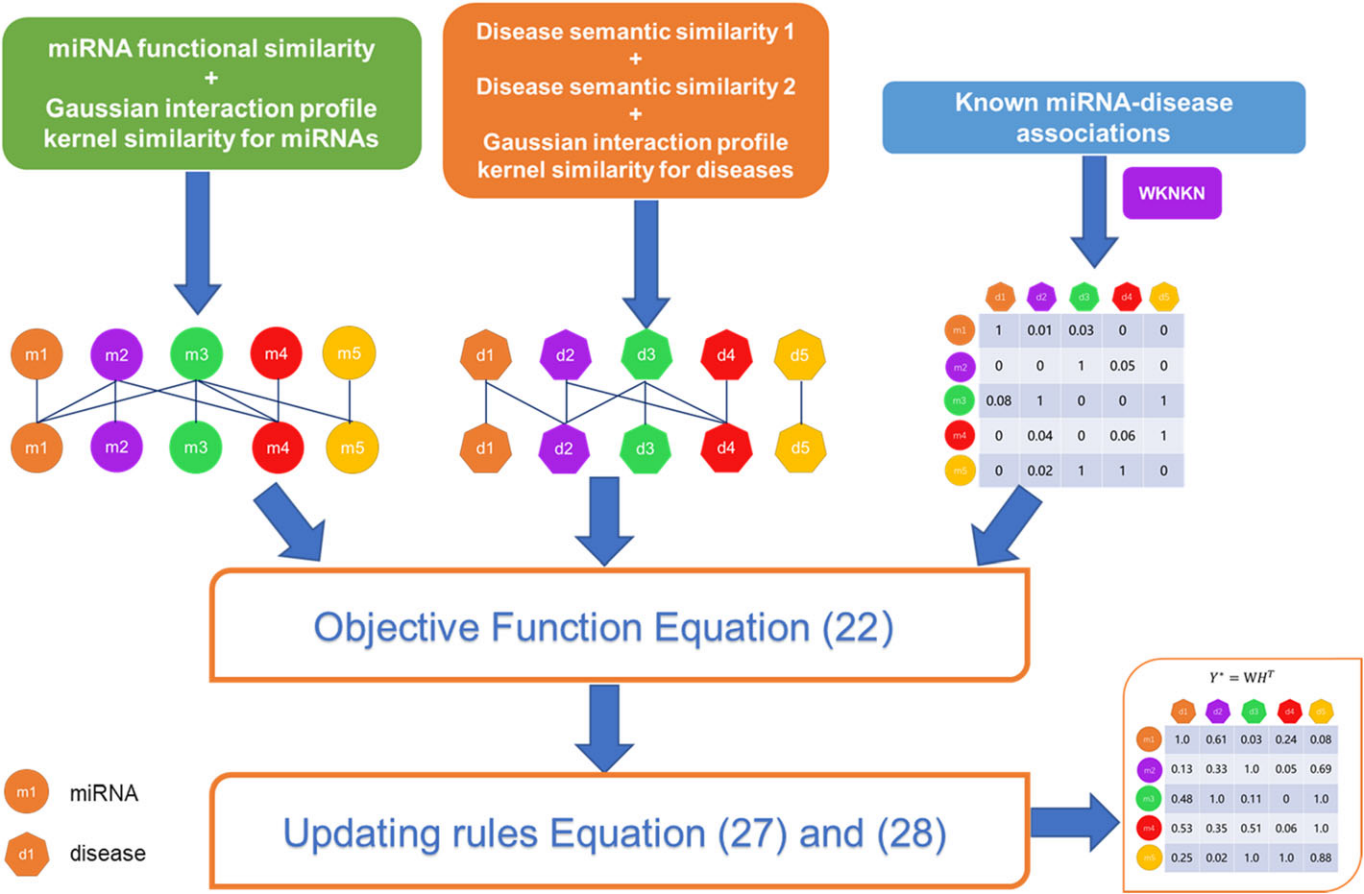
Flow chart of GRL2,1-NMF

Here, a new nonnegative matrix factorization method was presented to identify underlying miRNA-disease connections. The flow chart of *GRL*_2, 1_-NMF is shown in Fig. 3. We incorporated Tikhonov (*L*_2_), graph Laplacian regularization terms and the *L*_2, 1_-norm into the traditional NMF model for predicting miRNA-disease connections. The Tikhonov regularization is utilized to penalize the non-smoothness of *W* and *H* [48, 54, 55], and the graph Laplacian regularization is primarily intended to ensure local-based representation by leveraging the geometric structure of the data [56]. The *L*_2, 1_-norm was added to increase the disease matrix sparsity and eliminate unattached disease pairs [30, 52, 53]. The optimization problem of *GRL*_2, 1_-NMF can be formularized as follows:

22 $$ {\displaystyle \begin{array}{c}{\mathit{\min}}_{W,H}{\left\Vert Y-W{H}^T\right\Vert}_F^2+{\lambda}_l\left({\left\Vert W\right\Vert}_F^2+{\left\Vert H\right\Vert}_F^2\right)+{\lambda}_l{\left\Vert H\right\Vert}_{2,1}\\ {}+{\lambda}_m Tr\left({W}^T{L}_mW\right)+{\lambda}_d Tr\left({H}^T{L}_DH\right)\\ {}s.t.W\ge 0,H\ge 0\ \end{array}} $$

where ‖∙‖_*F*_ represents the Frobenius norm of a matrix; ‖·‖_2, 1_ represents the *L*_2, 1_-norm; Tr(∙) denotes the trace of a matrix; and *λ*_*l*_, *λ*_*m*_ and *λ*_*d*_ are regularization coefficients. Let *S*^*m*^ and *S*^*d*^ be miRNA and disease similarity networks; and let *D*_*m*_ and *D*_*d*_ be the diagonal matrices whose elements are row element or column element sums of *S*^*m*^ and *S*^*d*^ respectively. We define *L*_*m*_ = *D*_*m*_ − *S*^*m*^ and *L*_*d*_ = *D*_*d*_ − *S*^*d*^ as the graph Laplacian matrices for *S*^*m*^ and *S*^*d*^ [72], respectively; the first item denotes the similar matrix of the model for the purpose of searching for the matrices *W* and *H*. The next term is the Tikhonov regularization. The third item introduces the *L*_2, 1_-norm into matrix *H*. The last two items refer to the graph regularization of microRNAs and diseases.

### Optimization

Considering the two nonnegative constraints of the objective function, namely, *W* ≥ 0 and *H* ≥ 0, we utilized Lagrange multipliers to address the optimization problem in Equation (22). First, the Lagrange function *L*_*f*_ is as follows:

23 $$ {\displaystyle \begin{array}{c}{L}_f= Tr\left(Y{Y}^T\right)-2 Tr\left( YH{W}^T\right)+ Tr\left(W{H}^TH{W}^T\right)\\ {}\begin{array}{c}+{\lambda}_l Tr\left(W{W}^T\right)+{\lambda}_l Tr\left(H{H}^T\right)+{\lambda}_l{\left\Vert H\right\Vert}_{2,1}\\ {}+{\lambda}_m Tr\left({W}^T{L}_mW\right)+{\lambda}_d Tr\left({H}^T{L}_dD\right)H\\ {}+ Tr\left(\varnothing {W}^T\right)+ Tr\left(\varphi {H}^T\right)\end{array}\end{array}} $$

The partial derivatives of the above functions *L*_*f*_ for *W* and *H* are:

24 $$ \frac{\partial {L}_f}{\partial W}=-2 YH+2W{H}^TH+2{\lambda}_lW+2{\lambda}_m{L}_mW+\varnothing $$

25 $$ \frac{\partial {L}_f}{\partial H}=-2{Y}^TW+2H{W}^TW+2{\lambda}_lH+2{\lambda}_l AH+2{\lambda}_d{L}_d\mathrm{H}+\varphi $$

where *A* is a diagonal matrix, and the formula is as follows:

26 $$ {\left[A\right]}_{i,j}=\raisebox{1ex}{$1$}\!\left/ \!\raisebox{-1ex}{${\left\Vert {H}^s\right\Vert}_2$}\right.=\raisebox{1ex}{$1$}\!\left/ \!\raisebox{-1ex}{$\sqrt{\sum \limits_{j=1}^m{\left|{H}_{s,j}\right|}^2}$}\right. $$

Therefore, we obtained the updating rules expressed as Equations (27) and (28):

27 $$ {w}_{ik}\leftarrow {w}_{ik}\frac{{\left( YH+{\lambda}_m{S}^mW\right)}_{ik}}{{\left(W{H}^TH+{\lambda}_lW+{\lambda}_m{D}_mW\right)}_{ik}} $$

28 $$ {h}_{ik}\leftarrow {h}_{ik}\frac{{\left({Y}^TW+{\lambda}_d{S}^dH\right)}_{ik}}{{\left(H{W}^TW+{\lambda}_lH+{\lambda}_l AH+{\lambda}_d{D}_dH\right)}_{ik}} $$

According to Equation (27) and Equation (28), the nonnegative matrices *W* and *H* are updated until convergence. Eventually, we obtained a matrix of *Y*^∗^ = W*H*^*T*^, which is based on interactions among microRNAs and disease. We ranked predicted disease-connected miRNAs according to the elements in matrix *Y*^∗^. In theory, the higher-ranking miRNAs in each column of *Y*^∗^ tend to be connected with the matching disease.

## Supplementary information

**Additional file 1.** disease semantic similarity1.txt. This is disease semantic similarity 1, which integrated 383 disease semantic similarities.

**Additional file 2.** disease semantic similarity2.txt. This is disease semantic similarity 2, which integrated 383 disease semantic similarities.

**Additional file 3.** miRNA functional similarity.txt. This is the miRNA functional similarity, which integrated 495 miRNA functional semantic similarities.

**Additional file 4.** knownassociation.txt. This is a known miRNA-disease association matrix that was downloaded from HMDD v2.0. It includes 5430 known miRNA-disease associations between 495 miRNAs and 383 diseases.

**Additional file 5.** diseases_list.xlsx. This file lists 383 disease names.

**Additional file 6.** miRNAs_list.xlsx. This file lists 495 miRNA names.

## Data Availability

The dataset(s) supporting the conclusions of this article is (are) included within the article (and its additional files).

## Abbreviations

5-CV: Five-fold cross validation
AUC: The area under the ROC curve
DAG: Directed acyclic graph
dbDEMC: Database of differentially expressed miRNAs in human cancers
FPR: False positive rate
GIP: GAUSSIAN interaction profiles
HMDD: Human microRNA disease database
LOOCV: Leave-one-out cross validation
miRNA: MicroRNA
NMF: Nonnegative matrix factorization
ROC: Receiver operating characteristic
TPR: True positive rate
WKNKN: Weighted K nearest known neighbours
